# 738 Access to Burn Care: A 10 Year Perspective of Burn Centers in the U.S.

**DOI:** 10.1093/jbcr/irad045.213

**Published:** 2023-05-15

**Authors:** Elle Lovick, Jeffrey Carter, Herbert Phelan, Randy Kearns, Bart Phillips, William Hickerson

**Affiliations:** University Medical Center- New Orleans Burn Unit, New Orleans, Louisiana; University Medical Center New Orleans & LSUHSC, River Ridge, Louisiana; LSUHSC New Orleans Department of General Surgery, New Orleans, Louisiana; University of New Orleans, New Orleans, Louisiana; BData Inc, Minneapolis, Minnesota; Access Pro Medical, LLC, Augusta, Georgia; University Medical Center- New Orleans Burn Unit, New Orleans, Louisiana; University Medical Center New Orleans & LSUHSC, River Ridge, Louisiana; LSUHSC New Orleans Department of General Surgery, New Orleans, Louisiana; University of New Orleans, New Orleans, Louisiana; BData Inc, Minneapolis, Minnesota; Access Pro Medical, LLC, Augusta, Georgia; University Medical Center- New Orleans Burn Unit, New Orleans, Louisiana; University Medical Center New Orleans & LSUHSC, River Ridge, Louisiana; LSUHSC New Orleans Department of General Surgery, New Orleans, Louisiana; University of New Orleans, New Orleans, Louisiana; BData Inc, Minneapolis, Minnesota; Access Pro Medical, LLC, Augusta, Georgia; University Medical Center- New Orleans Burn Unit, New Orleans, Louisiana; University Medical Center New Orleans & LSUHSC, River Ridge, Louisiana; LSUHSC New Orleans Department of General Surgery, New Orleans, Louisiana; University of New Orleans, New Orleans, Louisiana; BData Inc, Minneapolis, Minnesota; Access Pro Medical, LLC, Augusta, Georgia; University Medical Center- New Orleans Burn Unit, New Orleans, Louisiana; University Medical Center New Orleans & LSUHSC, River Ridge, Louisiana; LSUHSC New Orleans Department of General Surgery, New Orleans, Louisiana; University of New Orleans, New Orleans, Louisiana; BData Inc, Minneapolis, Minnesota; Access Pro Medical, LLC, Augusta, Georgia; University Medical Center- New Orleans Burn Unit, New Orleans, Louisiana; University Medical Center New Orleans & LSUHSC, River Ridge, Louisiana; LSUHSC New Orleans Department of General Surgery, New Orleans, Louisiana; University of New Orleans, New Orleans, Louisiana; BData Inc, Minneapolis, Minnesota; Access Pro Medical, LLC, Augusta, Georgia

## Abstract

**Introduction:**

Only 2% of US hospitals have designated burn centers (BC), of which 57% are verified by the American Burn Association (ABA). Further, of the non-verified burn centers, it is unclear which ones meet requirements set by their state departments of health. This gap in knowledge of BC verification and/or designation status combined with the lack of an accurate, publicly available database to route patients and characterize BC resources creates challenges in burn care, especially in disaster preparedness. The goal of our study was to characterize the net effect of domestic BC openings and closings over the last decade on US burn resources and, thus, access to care.

**Methods:**

Our group has recently created the National Injury Resource Database (NIRD), a comprehensive list of every US Level I and II trauma center and/or burn center. NIRD was created by culling these facilities from 1) the current ABA burn center directory, 2) the current ABA burn center regional map, 3) the American Hospital Association database, 4) the American College of Surgeons Committee on Trauma database, and 5) data from all 50 State Departments of Health. Redundant entries were omitted, and 100% of the remaining institutions were then personally contacted by one of the study investigators to confirm or clarify the status of each center as well as the resources listed in the various databases. The NIRD database was then compared to the ABA’s 2012 burn center directory to compare the number, identities, and characteristics of those US BCs which had either closed or opened in the intervening ten years.

**Results:**

In 2012, there were 127 total BC, 63 of which were ABA verified. Currently, NIRD shows that there are 134 total BC recognized by the ABA, 76 of which are verified. Since 2012, 11 BC have been removed from the ABA’s burn center directory after either closing or failing to maintain ABA recognition, while 18 BC have been added. In the same time frame, there have been changes in BC verification status across the US, with 14 BC gaining ABA verification and 1 BC losing verification. In comparing the ABA’s current BC directory to their burn center regional map, we found variability in 18 centers across 15 states (CA, DC, IL, IN, KS, MD, ME, MI, MS, MO, NY, TN, TX, VA, VT), i.e., instances in which 1 or more BC found on the ABA’s BC regional map were not found on the ABA’s BC directory list.

**Conclusions:**

The last 10 years have shown an upward trend in the number of US BC. Further, we found variability in the ABA’s online resources in 15 states, suggesting that their resources are outdated. NIRD can serve as a resource for the construction of publicly available real-time tools to aid in routing burn patients.

**Applicability of Research to Practice:**

NIRD can serve as a resource for the construction of publicly available real-time tools to aid in routing burn patients.

